# Birth of a Live Cria After Transfer of a Vitrified-Warmed Alpaca (*Vicugna pacos*) Preimplantation Embryo

**DOI:** 10.3389/fvets.2020.581877

**Published:** 2020-12-03

**Authors:** Jennifer C. Lutz, Susan L. Johnson, Kimberly J. Duprey, Paul J. Taylor, Henry William Vivanco-Mackie, Daniel Ponce-Salazar, Marlene Miguel-Gonzales, Curtis R. Youngs

**Affiliations:** ^1^Cas-Cad-Nac Farm, Perkinsville, VT, United States; ^2^GeneSearch, Inc., Bozeman, MT, United States; ^3^Vivanco International SAC, Lima, Peru; ^4^Animal Science Department, Iowa State University, Ames, IA, United States

**Keywords:** cryopreservation, galactose, South American camelid, vitrification, hatched blastocyst

## Abstract

The alpaca (*Vicugna pacos*) is an important species for the production of fiber and food. Genetic improvement programs for alpacas have been hindered, however, by the lack of field-practical techniques for artificial insemination and embryo transfer. In particular, successful techniques for the cryopreservation of alpaca preimplantation embryos have not been reported previously. The objective of this study was to develop a field-practical and efficacious technique for cryopreservation of alpaca preimplantation embryos using a modification of a vitrification protocol originally devised for horses and adapted for dromedary camels. Four naturally cycling non-superovulated Huacaya females serving as embryo donors were mated to males of proven fertility. Donors received 30 μg of gonadorelin at the time of breeding, and embryos were non-surgically recovered 7 days after mating. Recovered embryos (*n* = 4) were placed individually through a series of three vitrification solutions at 20°C (VS1: 1.4 M glycerol; VS2: 1.4 M glycerol + 3.6 M ethylene glycol; VS3: 3.4 M glycerol + 4.6 M ethylene glycol) before loading into an open-pulled straw (OPS) and plunging directly into liquid nitrogen for storage. At warming, each individual embryo was sequentially placed through warming solutions (WS1: 0.5 M galactose at 37°C; WS2: 0.25 M galactose at 20°C), and warmed embryos were incubated at 37°C in 5% CO_2_ in humidified air for 20–22 h in 1 ml Syngro® holding medium supplemented with 10% (v/v) alpaca serum to perform an initial *in vitro* assessment of post-warming viability. Embryos whose diameter increased during culture (*n* = 2) were transferred individually into synchronous recipients, whereas embryos that did not grow (*n* = 2) were transferred together into a single recipient to perform an *in vivo* assessment of post-warming viability. Initial pregnancy detection was performed ultrasonographically 29 days post-transfer when fetal heartbeat could be detected, and one of three recipients was pregnant (25% embryo survival rate). On November 13, 2019, the one pregnant recipient delivered what is believed to be the world's first cria produced from a vitrified-warmed alpaca embryo.

## Introduction

The alpaca (*Vicugna pacos*) is a member of the South American Camelid (SAC) family consisting of alpacas, llamas, vicuñas, and guanacos. The SACs, which number more than 9.1 million head globally (1), convert low-quality roughages into fiber and meat in a variety of different production and management conditions. Particularly in parts of South America, alpacas are often a major source of income for farmers in remote rural areas (2).

In contrast with other livestock species such as cattle and sheep, alpaca genetic improvement programs seldom utilize artificial insemination or embryo transfer because those reproductive biotechnologies are not yet well-developed for alpacas (3, 4). Of special note with respect to embryo transfer technology is the difficulty in cryopreserving alpaca embryos (5) using either slow cooling or vitrification methods (6). Possible reasons for the difficulty in cryopreserving alpaca preimplantation embryos include the high lipid content of cells comprising the embryo (7) and the large number of cells present when embryos enter the uterus as hatched blastocysts (8). This challenge with cryopreservation of large diameter embryos is not unique to alpacas, as large diameter equine blastocysts have proven challenging to cryopreserve, especially using slow cooling techniques (9).

Pregnancies have been reported from the transfer of alpaca preimplantation embryos cryopreserved via slow cooling (10, 11) or vitrification (10). However, no live births were reported from those studies. The overall objective of this study was to develop a field-practical and efficacious protocol for vitrification of alpaca preimplantation embryos. The specific objective was to test a modification of a vitrification protocol originally devised for horses (12, 13) and modified for use in dromedary camels (14) because this protocol had proven successful in two different species—one of which is a relative of the alpaca (15).

## Materials and Methods

### Experimental Animals and Their Management

This field study was conducted at a privately owned Huacaya alpaca farm near Perkinsville, Vermont, USA (43.3737° N latitude and 72.5137° W longitude; elevation of 175 m above sea level). The coldest month is January (mean temperature of −7.8°C), and the warmest month is July (mean temperature of 20.3°C). Average annual precipitation is 1,022 mm. Animals were managed in groups of ~20 females in indoor pens measuring ~42 m^2^ with access to outside grazing pastures. Animals were hand-fed once daily a commercial 15% crude protein pellet (Poulin Grain Alpaca Milk & Cria Pellet, Newport, Vermont, USA). Animals had *ad libitum* access to fresh water, orchard grass hay, and a mineral mix specially formulated for alpacas (Stillwater Mineral Formula 104, Paoloa, Kansas, USA).

### Donor Selection

Embryo donors were evaluated for ovarian follicular development using a 7.5-MHz ultrasound transducer (Aloka SSD-500V). Females with ovarian follicles 7–10 mm in diameter were behavior tested to determine their sexual receptivity (16), and receptive females were naturally mated to a male alpaca of proven fertility. At the time of breeding (Day 0), donors (*n* = 4) were given 30 μg gonadorelin (Factrel®, Zoetis, Kalamazoo, MI USA) intramuscularly to aid in the induction of ovulation.

### Recipient Selection

Potential recipient females (*n* = 12) were evaluated ultrasonographically as described above. Females with ovarian follicles 7–10 mm in diameter were behavior tested, and sexually receptive females (*n* = 5) received 30 μg gonadorelin 6 days prior to embryo transfer. Final selection of suitable recipients was based on ultrasonographic confirmation of a corpus luteum and non-receptive behavior when exposed to a male 24 h prior to transfer on Day 6. We chose to perform 1-day asynchronous transfers (17, 18). Pregnancy testing of recipients was performed via transrectal ultrasonography (19) 29 days post-transfer (to visualize fetal heartbeat) and again at 52, 70, 84, 109, and 177 days post-transfer (to monitor the pregnancy and to enable the determination of the approximate timing of fetal death loss were it to occur).

### Media Preparation

Alpaca serum was prepared by collection of whole blood from four non-pregnant females at unknown stages of the estrous cycle into a 20-cc syringe. Blood was transferred into red-top Vacutainer® tubes (BD, Franklin Lakes, New Jersey, USA) before centrifugation at 3500 RPM for 20 min. Serum was sterile-filtered using a 0.22-μm low-protein binding Acrodisc® filter. Fresh serum was used to prepare the base medium and the culture medium. The culture medium and excess serum were frozen at −29°C until needed; the culture medium was thawed by placement onto a slide warmer at 37°C.

Glycerol and ethylene glycol were obtained from Fisher Scientific Company. All other chemicals were obtained from Sigma Aldrich Chemical Company. The base medium (BM) consisted of Dulbecco's phosphate buffered saline (D-PBS) supplemented with 0.3 mM sodium pyruvate, 3.3 mM galactose, and 20% (v/v) alpaca serum. Three vitrification solutions (VS) and two warming solutions (12–14) were subsequently prepared using BM. Vitrification solution 1 (VS1) was 1.4 molar (M) glycerol, VS2 was 1.4 M glycerol + 3.6 M ethylene glycol, and VS3 was 3.4 M glycerol + 4.6 M ethylene glycol. Warming solution 1 (WS1) was 0.5 M galactose, and WS2 was 0.25 M galactose. The culture medium consisted of Syngro® holding medium (Vetoquinol USA, Ft. Worth, Texas) supplemented with 10% (v/v) alpaca serum.

### Embryo Collection, Processing, and Transfer

Embryos were non-surgically collected 7 days postbreeding via uterine lavage using a 14-Fr 5cc Foley catheter. Harvested embryos were transferred from the room temperature flushing medium (ViGRO™ Complete Flush, Vetoquinol USA, Ft. Worth, Texas) to an embryo holding medium, and their diameter was measured using an eyepiece reticle that had been inserted into one eyepiece of a stereomicroscope and calibrated with a 25-mm stage micrometer (Klarmann Rulings, Inc., Litchfield, New Hampshire, USA). After measurement of the diameter, embryos were washed six times in holding medium supplemented with 10% (v/v) alpaca serum. Embryos were handled at 20°C unless otherwise noted.

Embryos were moved individually through a series of VS in 35-mm petri dishes: 500 μl drops of VS1 for 5 min, 500 μl drops of VS2 for 5 min, 5 μl drops of VS3 for 20 s, and 5 μl drops of VS3 for ≤20 s, while embryos were loaded into an open-pulled straw (OPS; Minitube USA, Verona, Wisconsin). Each OPS was plunged directly into liquid nitrogen where it was stored until recipient females were available (29 days).

At warming, each OPS was removed from liquid nitrogen, and its tip was submerged into a 1-ml drop of WS1 at 37°C. The embryo was expelled from the OPS into WS1 where it remained for 1 min before transfer into WS2 for 5 min at room temperature (20°C). Embryos were then transferred into a holding medium augmented with 10% (v/v) alpaca serum, and after 5 min, the embryo size was measured. Embryos were subsequently cultured at 37°C in 1 ml of culture medium in 4-well plates for 20–22 h in a gaseous environment of 5% CO_2_ in humidified air.

At the end of the culture period, embryo size was measured to enable assessment of embryo growth by comparing changes in the embryo size from the post-collection to the post-warming to the post-incubation measurement periods. Embryos were loaded individually into sterile 0.25-ml straws and placed into a conventional bovine embryo transfer gun, covered with a sterile sheath and chemise, for non-surgical embryo transfer. Embryos were transferred transcervically to the uterine horn ipsilateral to the ovary possessing a corpus luteum (17, 18).

### DNA Testing for Parentage Verification

On the private farm where this field study was conducted, it is standard operating procedure to perform DNA analysis on all animals to be registered in the alpaca breed association (Alpaca Owners Association, Inc.; formed from the merger of the Alpaca Owners & Breeders Association, Inc. and the Alpaca Registry, Inc.). Due to contractual agreements, the Alpaca Registry, Inc. and the Alpaca Owners Association, Inc. used different commercial DNA testing laboratories.

The DNA testing of the donor female was performed in November 2006 by the University of California–Davis veterinary genetics laboratory (Davis, California, USA). A whole blood sample was collected using a 3-cc syringe and then transferred into a 2-ml lavender top blood collection tube containing liquid EDTA as an anticoagulant. The blood tube was mailed to The Alpaca Registry, Inc. who subsequently forwarded it to the commercial DNA testing laboratory for analysis.

The DNA testing of the breeding sire and the recipient female was performed in January 2016 by DDC DNA Diagnostics Center (Fairfield, Ohio, USA), and the same laboratory performed DNA analysis of the cria produced after transfer of a vitrified-warmed embryo in November 2019. Blood samples were collected using a 3-cc syringe, and a few drops of blood were placed on a Whatman® FTA® card (Cytiva, Little Chalfont, UK) and allowed to dry before shipment via mail to the commercial DNA testing laboratory.

## Results

One embryo was recovered from each of the four donors, and all recovered embryos were hatched blastocysts with a mean diameter of 412 ± 62 μm (range of 275–575 μm). Embryo diameter was also measured post-warming and post-incubation/pre-transfer (Table 1). One embryo failed to increase in diameter during *in vitro* culture, while the remaining embryos had a minor (<25%), moderate (>25%, but <50%), or large (>50%) increase in volume during incubation.

**Table 1:** Size (in μm) of alpaca preimplantation embryos (*n* = 4) measured post-collection, post-warming/pre-incubation, and post-culture/pre-transfer.

**Donor** **ID tag**	**Post-collection (fresh embryo)**	**Post-warming**	**Post-culture/** **pre-transfer**	**Recipient** **ID tag**	**Uterine horn of embryo deposition^**a**^**	**Pregnancy outcome**
687	375 × 400	350 × 375	725 × 775	1505	Left	Pregnant
943	550 × 575	575 × 575	625 × 675	1356	Left	Not pregnant
1615	275 × 275	200 × 200	200 × 250	1038^b^	Right	Not pregnant
1501	325 × 400	300 × 350	300 × 350			

a *In all instances, embryos were transferred non-surgically to the uterine horn ipsilateral to the ovary that possessed a corpus luteum*.

b *This recipient received two embryos*.

Embryos that grew during culture (*n* = 2) and that were considered highly viable based on their growth during *in vitro* culture were transferred individually into synchronous recipients, whereas embryos whose post-culture diameter was similar or exhibited a minor increase in their pre-vitrification diameter (*n* = 2) were transferred together into a single recipient (with the hopes of sending a stronger signal for maternal recognition of pregnancy).

Among the three recipients, only one pregnancy was detected (in the recipient that received the vitrified-warmed embryo which exhibited a large amount of growth in culture post-warming). The overall embryo survival rate was 25% (one of four embryos).

**Figure 1:**
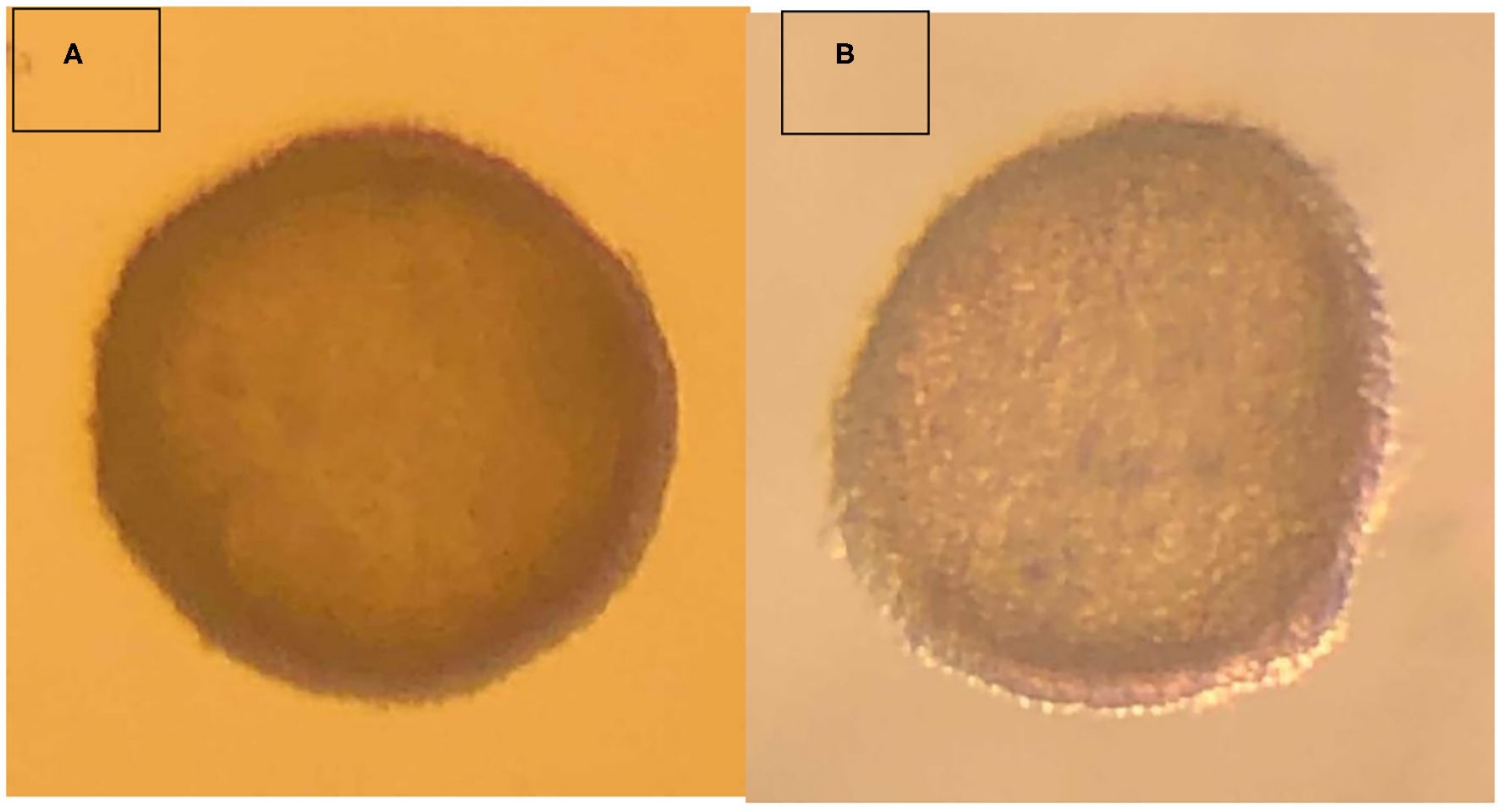
Vitrified-warmed alpaca preimplantation embryo that resulted in the birth of a live cria. **(A)** depicts the freshly collected embryo, and **(B)** depicts the embryo post-warming.

**Figure 2:**
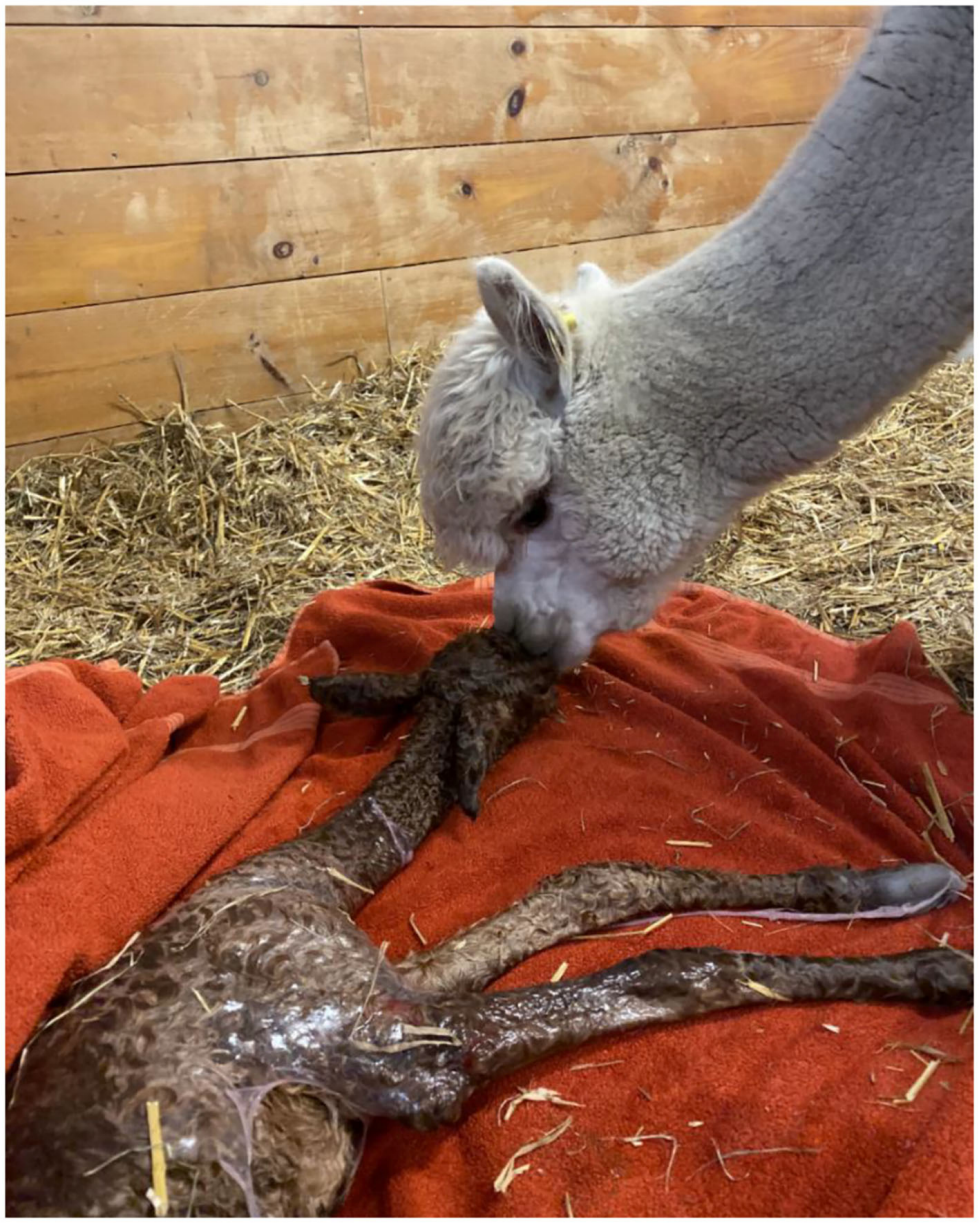
Recipient female and cria resulting from the transfer of a vitrified-warmed alpaca preimplantation embryo. The cria was born November 13, 2019.

**Figure 3:**
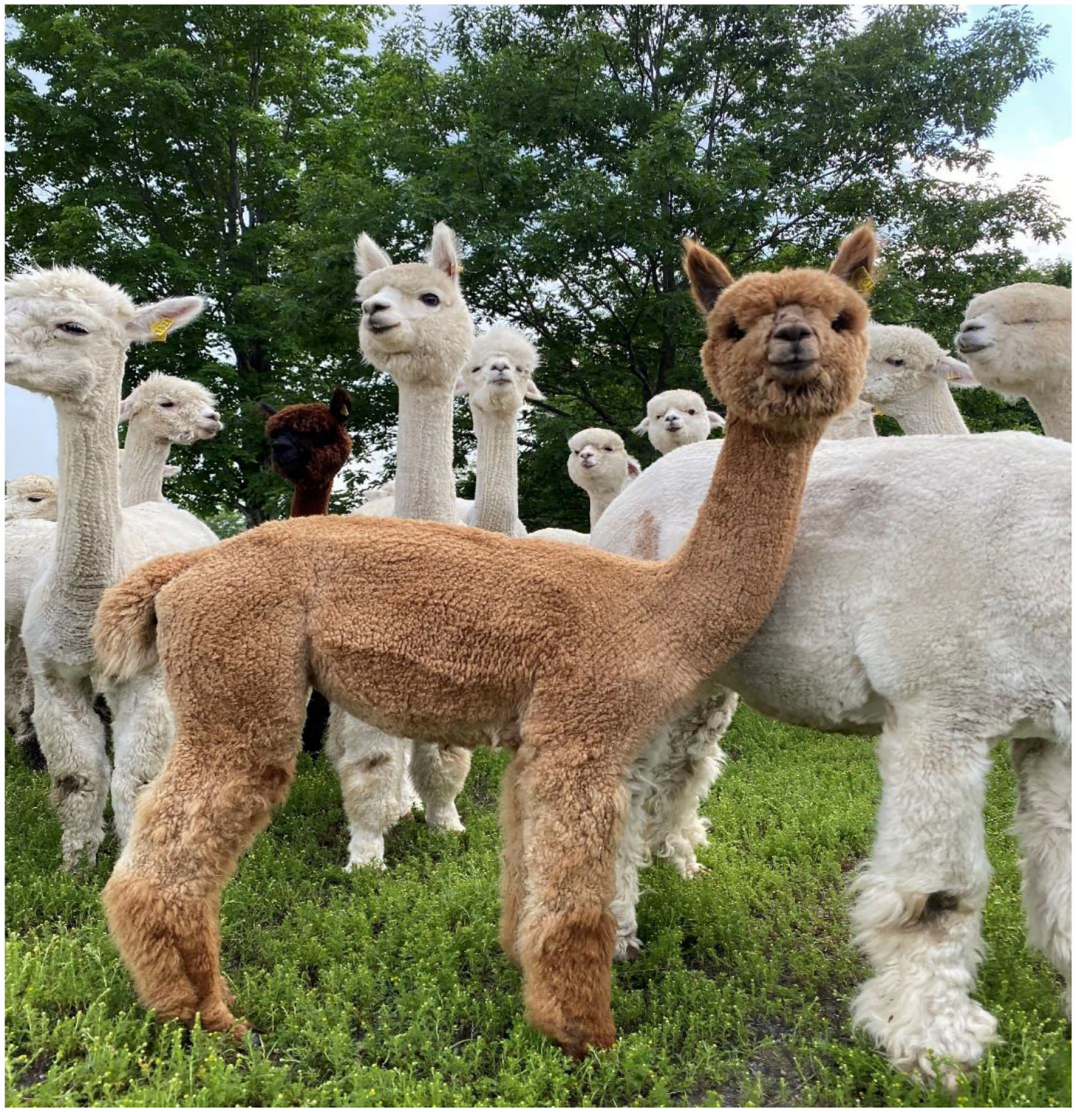
Cria (medium fawn color, in foreground) produced from a vitrified-warmed alpaca preimplantation embryo at ~7.5 months of age, demonstrating the normalcy of postnatal development.

The embryo (Figure 1) that nearly doubled in size during *in vitro* culture gave rise to a viable offspring. On November 13, 2019, a healthy female cria (Figure 2) weighing 8.1 kg was born after a gestation of 353 days. Parentage of the embryo transfer cria was confirmed through DNA testing (Table 2). The cria has grown normally and, at 7.5 months of age, is free of any detectable abnormalities (Figure 3).

**Table 2:** DNA verification of the parentage of the cria produced from a vitrified-warmed embryo.

	**Donor female^a^**		**Breeding sire^b^**		**Recipient female^b^**		**Cria^b^**	
**DNA marker**	**A1^**c**^**	**A2**	**A1**	**A2**	**A1**	**A2**	**A1**	**A2**
LCA5	192^d^	202	190	202	202	202	202	202
LCA8	231	241	237	259	237	243	237	241
LCA19	102	112	100	102	102	102	102	102
LCA37	n.d.	n.d.	134	134	134	158	134	156
LCA56	n.d.	n.d.	202	202	**198**^**e**^	**200**	202	226
LCA66	226	236	227	236	227	230	227	236
LPAC3	313	313	313	313	313	323	313	313
LPAC9	286	290	292	292	286	290	290	292
LPAC18	n.d.	n.d.	266	274	**268**	**268**	274	284
LPAC23	124	124	124	124	124	128	124	124
LPAC25	129	131	123	131	123	129	123	131
LPAC39	284	284	290	297	**285**	**293**	284	297
VOLP32	n.d.	n.d.	211	245	231	245	211	245
YWLL08	142	180	128	168	**136**	**160**	142	168
YWLL29	219	227	219	219	217	219	219	227
YWLL36	150	150	150	150	**164**	**170**	150	150
YWLL40	188	188	180	186	180	188	186	188

a *DNA testing of donor female performed by the University of California–Davis Veterinary Genetics Laboratory in November 2006*.

b *DNA testing performed by DDC DNA Diagnostics Center in January 2016, January 2016, and November 2019 for the breeding sire, recipient female, and cria, respectively*.

c *A1 denotes the allele inherited from one parent, whereas A2 denotes the allele inherited by the other parent*.

d *The number for each DNA marker represents identification of the specific allele; n.d. denotes a DNA marker that was not detected because it was not a part of the testing panel*.

e *Recipient female DNA markers LCA56, LPAC18, LPAC39, YWLL08, and YWLL36 (shown in bold font) demonstrate that the cria is genetically unrelated to the recipient*.

## Discussion

To the authors' knowledge, this birth represents the world's first cria produced after the transfer of a cryopreserved alpaca embryo. Preimplantation embryos recovered non-surgically from the uterus of alpaca donor females typically are hatched blastocysts > 400 μm in diameter (8), and this relatively large size poses a significant challenge to adequate removal of osmotically free water from the embryonic cells during the cryopreservation process. The high lipid content of alpaca embryos (7) also makes it difficult to achieve adequate embryo dehydration because the heads of phospholipids are hydrophilic.

Vitrification utilizes an ultrarapid cooling rate that causes intracellular water to transition from liquid to solid so quickly that ice crystal formation cannot occur (6). Vitrification has proven quite useful for large diameter equine blastocysts (13), as well as for high lipid content porcine (20) and dromedary camel embryos (21). We successfully employed a modified vitrification protocol to overcome the challenges to cryopreservation typically associated with large diameter and/or high lipid content embryos. The modifications we made to the previously published vitrification protocol (12–14) included the following: (1) replacement of glucose with galactose in the base medium, (2) replacement of fetal bovine serum with alpaca serum in all solutions containing serum, (3) use of a commercially available embryo holding medium for post-warming/pre-transfer temporary holding, (4) performing asynchronous embryo transfer (no more than 24 h asynchronous; standard operating procedure for embryo transfer on this farm) as is common in pigs (22) and horses (23), and (5) an overnight *in vitro* culture period to enable post-warming observation of greater duration (because embryos that initially appear viable shortly after warming often fail to produce pregnancies after embryo transfer); this enables a more accurate *in vitro* assessment of post-warming embryo viability.

We speculate that one or more of three factors likely contributed to our success in this study, although the small number of embryos used in this field study precludes an exact determination. Firstly, the vitrified-warmed embryo that produced the live cria was a relatively small hatched blastocyst (400 × 375 μm). Water removal from smaller diameter blastocysts should be easier than with larger diameter blastocysts (9). However, the effect of the size of camelid embryos on post-transfer pregnancy rate is unclear. In dromedary camels, embryos between 250 and 500 μm in diameter were more tolerant to vitrification than embryos <250 μm in diameter (24), yet embryo diameter had no effect in another study (21).

Secondly, we used galactose rather than glucose in the base medium used to prepare vitrification solutions, as had been done previously for bovine *in vitro* produced (25) and equine embryos (26). We also used galactose rather than sucrose as the non-permeating compound in the warming solutions as was done previously with equine (12, 13, 26), bovine *in vitro* produced (27), ovine (28), and dromedary camel embryos (14). The reasons for the greater apparent tolerance of alpaca embryos to the monosaccharide galactose vs. the disaccharide sucrose (14) are not known at present. Galactose and glucose are structural isomers (they have identical molecular formulas but different structures), whereas sucrose consists of glucose and fructose (a structural isomer of both galactose and glucose) held together by a glycosidic bond. Perhaps galactose is more beneficial than glucose in the initial pre-cooling dehydration of the embryo prior to vitrification. Alternatively, galactose may provide enhanced protection of cell membranes during vitrification and warming or may facilitate greater removal of permeating cryoprotectant from the embryo post-warming.

Thirdly, the recipient females in this study were housed in a herd that is accustomed to daily contact with humans. We believe that this low-stress environment may have enabled not only establishment of the pregnancy but also maintenance of the pregnancy to term. Previous work by several members of this same research team (10, 11) had resulted in several pregnancies with cryopreserved alpaca embryos; however, all pregnancies were lost within the first 4 months of gestation. Pregnancy loss in extensively managed alpacas is quite common and can range from 50 to 70% (29).

This report with alpacas follows a limited number of reports of successful cryopreservation of llama (30, 31) and dromedary camel embryos (21), as well as reports of pregnancies with frozen-thawed (32) and vitrified-warmed (33) alpaca embryos that apparently did not result in live births. In the llama research in Argentina (30), hatched blastocysts collected on Days 8.0–8.5 were vitrified in 0.25-ml straws in VS containing glycerol, ethylene glycol, sucrose, glucose, and polyethylene glycol (PEG). Eight vitrified-warmed embryos were transferred into four recipients, two of which became pregnant. Llama embryo cryopreservation research conducted in the United States (31) examined vitrification of Day 7 hatched blastocysts which had undergone pre-vitrification blastocoele cavity collapse and were subsequently vitrified in 0.25-ml straws using VS comprised of glycerol, butanediol, PEG, sucrose, and fetal bovine serum. The reduction of blastocoele fluid volume led to a higher pregnancy rate when compared with intact blastocysts. Those two llama studies differed from the present study in a multitude of ways: different VS, different WS, vitrification in straws vs. OPS, intact vs. collapsed blastocyst, and differences in post-warming incubation (0 or 2.5 h vs. 20–22 h in the present study). Dromedary research conducted in the United Arab Emirates (21) used a commercial camel embryo vitrification kit, presumably based on a previously published protocol (14), that was tested with or without bovine serum albumin supplementation. That study was comparable to ours, except that all warmed embryos were transferred as pairs. The 60-day pregnancy rate achieved was 37.5% (15 pregnancies/40 transfers), with an overall embryo survival rate of 19%.

The successful production of a live cria from the transfer of a vitrified-warmed alpaca embryo in this study gives great hope for further development and use of this reproductive biotechnology in genetic improvement programs for alpacas. We have produced a second live cria in Peru using the same protocol (Vivanco et al., unpublished data), which lends credence to our technique. Further research with a larger number of animals, however, is needed to ensure that our technique is robust.

## Data Availability Statement

The raw data supporting the conclusions of this article will be made available by the authors, without undue reservation.

## Ethics Statement

Ethical review and approval was not required for this animal study because embryo collection and transfer, which were implemented in 2011 at Cas-Cad-Nac Farm, are routine animal husbandry procedures on this farm. This project involved only client-owned animals, all animal-related work was done by the animal owner, and best practices for veterinary care were followed. Written informed consent was obtained from the owners for the participation of their animals in this study.

## Author Contributions

The idea for this experiment originated with JL. The experimental approach was developed by JL, CY, and PT using the methodology developed in part by HV-M, DP-S, and MM-G. The experiment was conducted by JL, SJ, and KD. The manuscript was written and/or edited by all authors.

## Conflict of Interest

The authors declare that this research was financed by and conducted at Cas-Cad-Nac Farm. JL, SJ, and KD are affiliated with or employed by Cas-Cad-Nac Farms. PT is affiliated with GeneSearch, Inc. HV-M, DP-S, and MM-G are affiliated with or employed by Vivanco International SAC. Although these results directly benefit Cas-Cad-Nac Farm, GeneSearch, Inc, and Vivanco International SAC, these results also benefit alpaca breeders throughout the world because they are being published in an open access journal. The remaining author declares that the research was conducted in the absence of any commercial or financial relationships that could be construed as a potential conflict of interest.
